# Artificial intelligence in advanced gastric cancer: a comprehensive review of applications in precision oncology

**DOI:** 10.3389/fonc.2025.1630628

**Published:** 2025-08-19

**Authors:** Min Fu, Jialing Xu, Yingying Lv, Baijun Jin

**Affiliations:** Department of Medical Oncology, The First People’s Hospital of Xiaoshan District, Hangzhou, China

**Keywords:** artificial intelligence, advanced gastric cancer, precision oncology, treatment response prediction, multi-modal data

## Abstract

Gastric cancer (GC) remains a major global health challenge, particularly in its advanced stages where prognosis is poor, and treatment responses are heterogeneous. Precision oncology aims to tailor therapies, but current biomarkers have limitations. Artificial Intelligence (AI), encompassing machine learning (ML) and deep learning (DL), offers powerful tools to analyze complex, multi-dimensional data from advanced GC patients, including clinical records, genomics, imaging (radiomics), and digital pathology (pathomics). This review synthesizes the current state of AI applications in unresectable, advanced GC. AI models demonstrate significant potential in refining diagnosis and staging, predicting treatment efficacy for chemotherapy, immunotherapy, and targeted therapies, and assessing prognosis. Multi-modal AI approaches, integrating data from diverse sources, consistently show improved predictive performance over single-modality models, better reflecting the complexity of the disease. Key challenges remain, including data quality and standardization, model generalizability and interpretability, and the need for rigorous prospective validation. Future directions emphasize multi-center collaborations, development of robust and explainable AI (XAI), and seamless integration into clinical workflows. Overcoming these hurdles will be crucial to translate AI’s potential into tangible clinical benefits, enabling truly personalized and effective management for patients with advanced gastric cancer.

## 1 Introduction

Gastric cancer (GC) represents a significant global health burden, ranking as the fifth most common malignancy and a leading cause of cancer-related mortality worldwide (1). Despite advances in treatment, the prognosis for patients diagnosed with advanced or metastatic GC remains poor, with 5-year overall survival rates often falling below 30%, and in metastatic settings, below 10% (2). A substantial proportion of patients present with unresectable disease at diagnosis due to non-specific early symptoms. Standard therapeutic approaches, including systemic chemotherapy, targeted therapy for specific molecular subtypes (e.g., human epidermal growth factor receptor 2-positive tumors), and immunotherapy (immune checkpoint inhibitors, ICIs), form the backbone of management (3). However, patient responses to these treatments are highly heterogeneous, and many individuals derive limited benefit or develop resistance, highlighting a critical unmet need for more effective and personalized therapeutic strategies (4).

Precision oncology seeks to address this challenge by tailoring treatment strategies based on the unique characteristics of an individual patient’s tumor and host factors (5). This paradigm relies heavily on the identification and utilization of predictive biomarkers. In advanced GC, established biomarkers such as HER2 amplification, programmed death-ligand 1 (PD-L1) expression (often measured by Combined Positive Score, CPS), microsatellite instability-high (MSI-H) or deficient mismatch repair (dMMR) status, and more recently, claudin 18.2 (CLDN18.2) expression, guide the use of specific targeted therapies and immunotherapies (6). However, these biomarkers have inherent limitations. Their assessment often requires invasive tissue sampling, which may not be feasible or representative, especially in the metastatic setting. Interpretation of immunohistochemistry (IHC) can be variable, and genomic sequencing assays like next-generation sequencing (NGS) can be costly with significant turnaround times. Crucially, existing biomarkers often fail to capture the full spectrum of tumor heterogeneity and the complex interplay within the tumor microenvironment (TIME), leading to imperfect prediction of treatment response (7, 8).

Artificial Intelligence (AI), particularly its subfields of Machine Learning (ML) and Deep Learning (DL), offers a powerful set of tools to overcome these limitations. ML algorithms, such as Support Vector Machines (SVM), Random Forests (RF), and logistic regression, learn patterns from data to make predictions without explicit programming (9). DL models, including Convolutional Neural Networks (CNNs) and Artificial Neural Networks (ANNs), utilize hierarchical layers of processing units to automatically extract complex features and relationships from high-dimensional data. These capabilities make AI uniquely suited to analyze the vast and complex datasets generated in modern oncology, including clinical records, genomic and transcriptomic data (NGS), medical images (computed tomography, positron emission tomography, magnetic resonance imaging, endoscopy), and digitized histopathology slides (Whole Slide Images, WSI) (10, 11). The confluence of pressing clinical needs in advanced GC, the exponential growth in digital biomedical data, and the maturation of AI algorithms has fueled a rapid increase in research exploring AI’s potential in this field (12). While much early AI research in GC focused on improving endoscopic detection of early-stage disease, a significant opportunity lies in leveraging AI for the complex decision-making required in advanced stages. The profound tumor heterogeneity, diverse treatment options, and high stakes associated with advanced GC management present challenges where AI’s capacity for integrating multi-faceted data can offer substantial clinical value (13).

This review provides a comprehensive overview of the current applications of AI in the precision oncology landscape specifically for advanced, unresectable gastric cancer. It synthesizes evidence on the use of AI for refining diagnosis relevant to treatment selection, predicting treatment efficacy and toxicity, enabling personalized therapy through multi-modal data integration, and exploring novel strategies for managing refractory disease. The aim is to critically evaluate the current state-of-the-art, highlighting both the practical utility of emerging AI tools for clinicians and researchers and the key challenges and future directions necessary to translate these technologies into improved patient outcomes.

## 2 AI-driven precision diagnostics and staging refinement in advanced gastric cancer

Although the primary focus of this review is unresectable advanced GC, the accurate characterization and staging of the disease remain fundamental for determining prognosis and guiding the selection and intensity of systemic therapies. AI offers capabilities to extract deeper insights from standard diagnostic modalities, potentially refining risk stratification beyond traditional Tumor-Node-Metastasis (TNM) staging.

### 2.1 Radiomics for advanced GC characterization

Radiomics involves the high-throughput extraction and analysis of quantitative features from medical images (CT, PET, MRI), converting them into mineable data that can reveal underlying pathophysiology often invisible to the human eye (14). In advanced GC, radiomics models have shown promise in several areas. AI algorithms, particularly CNNs and Faster Region-based Convolutional Neural Networks (FR-CNN), have been developed to identify preoperative peritoneal metastasis from CT images (15). Predicting lymph node metastasis (LNM) is another critical application, as conventional CT imaging has known limitations, especially after neoadjuvant therapy which can alter lymph node morphology and size (16). Several studies have demonstrated that CT-based radiomics models, often employing ML algorithms like K-Nearest Neighbors (KNN), SVM, or DL approaches, can predict LNM status or quantify the number of metastatic nodes with significantly higher accuracy (Area Under the Curve [AUC] values often exceeding 0.75-0.80) compared to radiologists’ assessment alone (15). This improved LNM prediction, even in unresectable patients, provides valuable prognostic information that could influence decisions regarding the aggressiveness of palliative treatment or eligibility for clinical trials targeting specific metastatic patterns (13). Beyond metastasis detection, radiomics can quantify intra-tumoral heterogeneity, a known factor influencing treatment response and prognosis, by analyzing texture features within the tumor volume (17). Furthermore, AI applied to CT scans can perform automated body morphometry analysis, assessing features like sarcopenia (muscle wasting), which may correlate with patient frailty, treatment tolerance, and overall survival (18). The ability of radiomics to extract such detailed information non-invasively from standard-of-care imaging is a key advantage (19). Patients with advanced GC typically undergo repeated CT or PET scans for initial staging and treatment monitoring. Radiomics leverages this existing data to provide deeper biological insights without requiring additional invasive procedures, enabling longitudinal assessment of tumor characteristics and heterogeneity, which is crucial for adapting treatment in the dynamic advanced disease setting (20).

### 2.2 Radiomics for occult peritoneal metastasis detection

Peritoneal metastasis represents the most common pattern of distant metastasis in advanced GC, occurring in up to 66% of patients and serving as a major determinant of prognosis and treatment strategy selection (15, 20). The detection of occult peritoneal metastasis remains a critical clinical challenge, as patients with undetected peritoneal disease may undergo inappropriate surgical interventions or miss opportunities for optimal systemic therapy.

Conventional CT imaging demonstrates significant limitations in detecting early-stage peritoneal metastasis, with sensitivity as low as 28.3%-50.9%, particularly for tumor implants smaller than 1 cm (20, 21). These diagnostic limitations have led to the development of AI-driven approaches specifically targeting occult peritoneal metastasis detection. Dong et al. developed a pioneering radiomic nomogram incorporating both primary tumor and peritoneal region features, achieving excellent performance with AUCs of 0.958 in training and 0.928-0.941 in external validation cohorts across multiple centers (20). This approach demonstrated that both the tumor characteristics (“seed”) and peritoneal microenvironment features (“soil”) contribute to metastatic potential, reflecting the biological basis of the “seed and soil” hypothesis.

Building upon radiomics approaches, deep learning models have shown even greater promise. The Peritoneal Metastasis Network (PMetNet) developed by Jiang et al. achieved remarkable performance with AUCs of 0.946-0.920 in external validation, demonstrating sensitivities of 75.4%-87.5% and specificities of 92.9%-98.2% (22). Importantly, this model substantially outperformed conventional clinicopathological factors and could identify occult peritoneal metastasis missed by radiologist interpretation. The integration of gradient-weighted class activation mapping (Grad-CAM) provided interpretability by highlighting intra-tumoral regions associated with metastatic potential, suggesting that tumor heterogeneity may be a key factor in determining peritoneal spread.

Beyond imaging-based approaches, novel AI-assisted molecular techniques are emerging. Chen et al. developed stimulated Raman molecular cytology (SRMC), combining three-color stimulated Raman scattering microscopy with deep learning for analyzing exfoliated cells in peritoneal lavage fluid (23). This approach achieved 81.5% sensitivity and 84.9% specificity within 20 minutes, representing a significant advancement over conventional cytology’s <60% sensitivity. The method’s ability to analyze both cellular morphology and molecular composition (lipid, protein, and DNA content) demonstrates the potential for AI-driven integration of multiple biological features.

These advances in AI-powered peritoneal metastasis detection address a critical unmet clinical need in advanced GC management. The ability to accurately identify occult peritoneal disease preoperatively could significantly impact treatment decision-making, helping to avoid unnecessary surgical procedures while ensuring appropriate patients receive optimal systemic therapy or are considered for specialized peritoneal-directed treatments.

### 2.3 Pathomics for advanced GC characterization

Pathomics, or computational pathology, applies AI techniques to analyze digitized WSI obtained from tissue biopsies or surgical specimens (24). Even from small biopsy samples typically available in the advanced/metastatic setting, AI can extract a wealth of information. DL models, such as those based on ResNet or Inception architectures, have demonstrated high accuracy (often >90-95%) in classifying gastric tissue as normal, dysplastic, or cancerous, potentially aiding pathologists and reducing inter-observer variability (15). AI can also automate tasks like histological grading or classifying GC subtypes according to systems like the Lauren classification (25). A particularly promising application is the analysis of the TIME from H&E stained slides. AI algorithms can automatically detect and quantify TILs or assess stromal characteristics, features known to be associated with prognosis and response to immunotherapy (26). Furthermore, AI can assist in the interpretation of IHC staining for key biomarkers like HER2 and PD-L1. By objectively quantifying staining intensity and distribution, AI may improve the consistency and accuracy of biomarker assessment, which is critical for guiding targeted therapy and immunotherapy decisions (6). Similar to radiomics, pathomics offers a way to extract quantitative, objective data from routinely collected diagnostic materials (biopsy slides), providing insights into tumor biology and heterogeneity (27). This is valuable in advanced GC where metastatic tissue biopsies might be the only available samples, and understanding their characteristics is key to treatment planning.

## 3 AI for predicting treatment efficacy in advanced gastric cancer

A major goal of precision oncology in advanced GC is to predict which patients are most likely to benefit from a specific therapy, thereby maximizing efficacy while minimizing unnecessary toxicity and cost. AI is being extensively investigated for its potential to predict responses to various systemic treatments (**Table 1**).

**Table 1:** Summary of key AI studies predicting treatment efficacy in advanced gastric cancer.

Study	Patient Cohort (Size, Stage, Treatment)	AI Model Type	Input Data Modality(ies)	Prediction Task	Key Performance Metric(s)	Key Limitations/Notes	Ref.
Zhang (2024)	60 AGC, nICT	XGBoost (Radiomics), Nomogram (Radio+Clinical)	Baseline CT Radiomics, Clinical (PD-L1, cTNM, etc.)	Pathological Response (TRG) to nICT	Nomogram AUC: 0.893 (Test)	First radiomics study for nICT response; Small test set (n=18)	(20)
Liang (2022)	87 Advanced GC, Anti-PD-1	Radiomics Nomogram (Radio+Clinical)	CT Radiomics, Clinical	Response to Anti-PD-1	AUC: 0.865 (Train), 0.778 (Valid); Predicted PFS difference	Retrospective, single-center	(33)
Chen (2022)	286 AGC, NAC/Targeted	Radiomics (LASSO-LR)	Baseline CT Radiomics	Pathological Response (TRG) to NAC	AUCs: 0.72-0.82 across NAC cohorts; Poor for SOXA cohort (AUC 0.50)	Shows generalization for NAC but not targeted therapy; Retrospective	(28)
Li (2018)	58 LAGC, Preop Chemo	LDA Filter + RF (Radiomics)	Pre-treatment CT Radiomics (Portal Phase)	Pathological Response (GR vs non-GR)	AUC: 0.722 (PP)	Small sample size; Heterogeneity in methods	(4)
Xu (2021)	292 AGC, Neoadjuvant Chemo	Radiomics (LASSO-LR)	Baseline CT (Prediction), Restaging CT (Detection)	Pathological Downstaging (pDS)	Prediction AUC: 0.71 (Test I), 0.70 (Test II); Detection AUC: 0.74 (Test I), 0.76 (Test II)	Retrospective, specific chemo regimens (SEEOX/SOX)	(34)
Liu (2021)	69 AGC, Systemic Chemo	Multi-energy Radiomics Nomogram (Radio+Clinical)	Dual-Energy CT Radiomics (Multi-keV), Clinical (Stage, IC)	Clinical Response (RECIST) to Systemic Chemo	Nomogram AUC: 0.934	Retrospective, two centers, small sample size	(35)
Liu (2024)	264 Advanced GC, 1st Line PD-1 + Chemo	Ensemble DL (ICIsNet: EfficientNet, DenseNet, Swin Transformer)	Pre-treatment Biopsy WSI (H&E Pathomics)	Response (iRECIST) to 1st Line ICI+Chemo	WSI-level AUCs: 0.920 - 1.000 across 4 test cohorts	First study for 1st line ICI+Chemo prediction from biopsy; Retrospective	(23)
Li (2022)	58 Stage IV GC, ICI	Delta-Radiomics (LASSO-Cox)	Baseline & Follow-up CT Radiomics (Intra- & Peri-tumoral)	Progression-Free Survival (PFS) on ICI	ΔVintra_ZV feature predictive of PFS (HR 1.911, C-index 0.705)	Retrospective, small sample size	(31)
Zhou (2025)	429 HER2+ GC (310 Anti-HER2, 119 Anti-HER2+ICI)	Deep Learning (MuMo)	Radiology, Pathology, Clinical (Multi-modal)	Response to Anti-HER2 or Anti-HER2+ICI	AUC: 0.821 (Anti-HER2), 0.914 (Combined); Predicted survival difference	Multi-modal approach for specific subtypes/treatments; Retrospective	(32)
Jiang (2023)	2799 GC (Prognosis/Chemo); 303 Advanced GC (ICI)	Biology-Guided DL (BgDL - CNN)	Preoperative CT Imaging (linked to TME classes via IHC)	Prognosis (DFS), Chemo Benefit, ICI Response	Independent prognostic factor; Stratified chemo benefit; Predicted ICI response, complemented MMR/PD-L1	Links imaging to biology; International validation; Retrospective	(22)
Gao (2024)	2387 LAGC (10 centers) + 132 prospective, Neoadjuvant Chemo	Interpretable AI (iSCLM - Supervised Contrastive Learning)	Pre-treatment CT + Biopsy WSI (Multi-modal)	Pathological Response to Neoadjuvant Chemo	AUCs: 0.846-0.876 (Test cohorts)	Large multi-center study; Focus on interpretability (SHAP, heatmaps)	(29)

AGC, Advanced Gastric Cancer; AI, Artificial Intelligence; AUC, Area Under the Curve; CI, Confidence Interval; Clin, Clinical; CNN, Convolutional Neural Network; CT, Computed Tomography; DL, Deep Learning; DFS, Disease-Free Survival; EHR, Electronic Health Record; GR, Good Response; H&E, Hematoxylin & Eosin; HER2, Human Epidermal Growth Factor Receptor 2; HR, Hazard Ratio; ICI, Immune Checkpoint Inhibitor; iRECIST, Immune Response Evaluation Criteria In Solid Tumors; LAGC, Locally Advanced Gastric Cancer; LASSO, Least Absolute Shrinkage and Selection Operator; LDA, Linear Discriminant Analysis; LR, Logistic Regression; LNM, Lymph Node Metastasis; ML, Machine Learning; MMR, Mismatch Repair; MRI, Magnetic Resonance Imaging; MSI, Microsatellite Instability; NAC, Neoadjuvant Chemotherapy; nICT, Neoadjuvant Immunochemotherapy; NGS, Next-Generation Sequencing; ORR, Objective Response Rate; OS, Overall Survival; Patho, Pathomics; PD-1, Programmed Death-1; PD-L1, Programmed Death Ligand-1; PET, Positron Emission Tomography; PFS, Progression-Free Survival; pDS, Pathological Downstaging; Radio, Radiomics; RECIST, Response Evaluation Criteria In Solid Tumors; RF, Random Forest; SVM, Support Vector Machine; TME, Tumor Microenvironment; TRG, Tumor Regression Grade; WSI, Whole Slide Image; XGBoost, eXtreme Gradient Boosting.

### 3.1 Predicting response to systemic chemotherapy

Systemic chemotherapy remains a cornerstone of treatment for many patients with advanced GC, either in the palliative setting or potentially as neoadjuvant treatment for initially unresectable patients who might become candidates for conversion surgery. Predicting response is crucial for optimizing treatment selection. Numerous studies have explored the use of CT-based radiomics to predict pathological response (often assessed by Tumor Regression Grade, TRG) or clinical response (e.g., according to Response Evaluation Criteria in Solid Tumors, RECIST) to neoadjuvant or palliative chemotherapy (28). These studies employ various ML algorithms, including logistic regression (LR), SVM, RF, eXtreme Gradient Boosting (XGBoost), and DL models like DenseNet or V-Net, trained on features extracted from pre-treatment CT scans or changes observed between baseline and follow-up scans (delta-radiomics) (28). Reported performance metrics, such as AUC values, often range from 0.70 to over 0.85, suggesting a definite predictive signal. Some evidence suggests these radiomic signatures might generalize across different chemotherapy regimens, although inconsistencies in methodology and patient cohorts remain a challenge. Pathomics is also emerging as a predictive tool; for instance, interpretable AI frameworks like iSCLM (incremental Supervised Contrastive Learning Model), using supervised contrastive learning on pre-treatment biopsy WSIs combined with CT data, have achieved AUCs around 0.85 for predicting neoadjuvant chemotherapy response (29). Additionally, older studies explored ML models like SVM using clinical and demographic data, or gene expression profiles, to predict benefit from adjuvant chemotherapy, indicating the potential of non-imaging data as well (15). While the numerous radiomics studies reflect a strong clinical need for pre-treatment stratification, the variability in methods and often moderate predictive accuracy suggest that radiomics alone may not be sufficient for robust clinical decision-making. Its value likely lies in capturing tumor phenotypic information that complements other data modalities within integrated models.

### 3.2 Predicting response to immunotherapy (ICI)

ICIs, particularly anti-PD-1/PD-L1 antibodies, have become integral to the treatment of advanced GC, both as monotherapy in later lines and increasingly in combination with chemotherapy in the first-line setting (23). However, only a subset of patients responds, making predictive biomarkers essential. AI offers promising avenues to improve upon or complement existing biomarkers like PD-L1 CPS, TMB, and MSI/dMMR status, which have recognized limitations related to predictive accuracy and assessment challenges (30). Radiomics models based on CT or PET imaging features have been developed to predict response or survival outcomes in patients receiving anti-PD-1 therapy, with reported AUCs reaching up to 0.86. Delta-radiomics, assessing changes in imaging features during treatment, may also provide prognostic information (31). Pathomics approaches using DL on diagnostic H&E biopsy WSIs have shown remarkable success in predicting response to first-line PD-1 inhibitor plus chemotherapy combinations, achieving AUCs exceeding 0.90 in some validation cohorts (23). These models likely capture subtle morphological features of the tumor cells and the surrounding microenvironment (e.g., TIL density, stromal characteristics) that correlate with immune response. On the genomic front, AI algorithms like SVM have been used to analyze immuno-oncology related gene expression signatures from tumor samples to generate predictive scores (e.g., ‘ Immuno-Oncology Score’) for durable clinical benefit (21). Furthermore, innovative approaches like biology-guided deep learning (BgDL) explicitly link imaging features (from CT) to predicted TME subtypes (based on IHC markers like IS_GC_ and POSTN) to predict ICI response (22). Notably, such models may identify potential non-responders even within biomarker-positive groups like dMMR patients, suggesting a role in refining patient selection beyond current guidelines (22). The ability of AI to leverage non-invasive imaging or readily available biopsy slides offers a significant practical advantage over repeated invasive sampling, potentially providing a more comprehensive and dynamic assessment of immunotherapy response likelihood (23).

### 3.3 Predicting response to targeted therapies

Anti-HER2 therapy (e.g., trastuzumab) is standard for HER2-positive advanced GC, but response rates are not universal, and resistance can develop. While HER2 status determined by IHC/FISH is the primary selection criterion, AI may offer ways to refine prediction within this group. Research is exploring whether radiomic or pathomic features can capture tumor heterogeneity or other biological factors associated with response or resistance to anti-HER2 agents (32). Multi-modal AI models, such as the MuMo (Multi-Modal) model, integrate radiology, pathology, and clinical data to specifically predict response to anti-HER2 monotherapy (achieving an AUC of ~0.82) and, importantly, to the increasingly used combination of anti-HER2 therapy plus ICIs (AUC ~0.91) (32). The development of AI models specifically targeting combination therapies reflects the evolving treatment landscape and AI’s capacity to handle the increased complexity of predicting outcomes for multi-agent regimens, moving beyond single-agent prediction to potentially guide more complex treatment selections.

## 4 AI-powered multi-modal data integration for personalized treatment selection

The inherent complexity and heterogeneity of advanced GC necessitate a holistic understanding that often transcends the information available from any single data source (36). Clinicians naturally integrate diverse information—patient history, physical examination, laboratory results, imaging reports, pathology findings—to make treatment decisions. AI, particularly through multi-modal learning, aims to replicate and enhance this integrative process by computationally combining data from various modalities, such as clinical records, genomics/transcriptomics, radiomics (from CT, PET, MRI), and pathomics (from WSI) (36). This approach promises a more comprehensive patient profile, potentially leading to more accurate predictions and truly personalized treatment selection (37).

Several strategies exist for integrating multi-modal data within AI frameworks. Early fusion involves concatenating raw or processed features from different modalities before feeding them into a single model. Intermediate fusion learns separate representations for each modality first, then integrates these representations at one or more hidden layers within the model; this allows for more flexibility and can utilize techniques like attention mechanisms, gating mechanisms, graph neural networks (GNNs), or Kronecker products to model cross-modal interactions effectively (29). Late fusion involves training separate models for each modality and then combining their outputs or predictions (e.g., through averaging, voting, or meta-learning) to reach a final decision (37). The choice of fusion strategy often depends on the specific task, data types, and desired level of interaction modeling.

Numerous studies are now demonstrating the value of multi-modal AI in advanced GC. Integrating radiomic features derived from CT or PET scans with clinical variables (like TNM stage, performance status, lab values) has consistently shown improved performance in predicting survival or treatment response compared to using either modality alone, often reflected in higher C-indices or AUCs (13). Similarly, combining pathomic features from WSIs with clinical data has enhanced prognostic accuracy (37). More advanced integrations are also emerging. The iSCLM framework successfully combined pre-treatment CT scans and H&E biopsy images to predict neoadjuvant chemotherapy response with high accuracy across multiple centers (29). Radiopathomics models integrating features from both imaging and pathology slides are being developed for improved staging (38). Biology-guided approaches explicitly link imaging features to molecular or TME characteristics (derived from IHC or genomics) to predict outcomes, as seen in the BgDL model (22). Perhaps the most comprehensive examples involve integrating three or more modalities; the MuMo model effectively combined radiology, pathology, and clinical data to predict response to HER2-targeted therapy with or without immunotherapy (32), and other studies have integrated CT-derived radiomic and DL features with clinical variables for survival prediction (39). These studies consistently report that multi-modal models outperform their unimodal counterparts, underscoring the benefit of data integration (37). The increasing focus on such integrative models signals a maturation of the field, moving beyond single-modality proofs-of-concept towards approaches that better mirror the complexity of clinical reality and offer potentially more robust and relevant predictions (**Table 2**).

**Table 2:** Overview of multi-modal AI models in advanced gastric cancer.

Study	Model Name/Type	Input Modalities Integrated	Fusion Strategy	Application/ Prediction Task	Key Performance Metric(s)	Key Findings/Interpretability Aspects	Ref
Chen (2023)	AI-annotated Clinical-Pathologic Risk Model (XGBoost)	Clinical, Pathological	Feature Integration (likely early/intermediate)	Prognosis (Survival) in Metastatic GC	C-index: ~0.70-0.74 (Train/Test/External Valid)	Used SHAP for feature importance interpretation	(13)
Liang (2022)	Radiomics Nomogram	CT Radiomics, Clinical	Late Fusion (Nomogram)	Response to Anti-PD-1	AUC: 0.865 (Train), 0.778 (Valid); Outperformed clinical model	Combined imaging features with clinical risk factors	(33)
Wang (2019)	Radiomics Nomogram (LASSO Cox)	CT Radiomics (2D/3D), Clinical	Late Fusion (Nomogram)	Prognosis (OS) after Resection	Nomogram C-index: 0.82 (vs. 0.71 Radio, 0.74 Clin)	2D radiomics performed better than 3D in test set	(40)
Liu (2021)	Clinical-Radiomics Nomogram	Dual-Energy CT Radiomics, Clinical	Late Fusion (Nomogram)	Clinical Response (RECIST) to Systemic Chemo	Nomogram AUC: 0.934; Outperformed unimodal models	Multi-energy radiomics improved prediction	(35)
Zhou (2025)	MuMo (Deep Learning)	Radiology, Pathology, Clinical	Deep Learning Integration (details not specified)	Response to Anti-HER2 or Anti-HER2+ICI	AUC: 0.821 (Anti-HER2), 0.914 (Combined); Predicted survival	Addresses specific combination therapy; Interpretability via Grad-CAM/feature importance	(32)
Jiang (2023)	BgDL (CNN)	CT Imaging, TME status (derived from IHC)	Biology-Guided Intermediate Fusion	Prognosis (DFS), Chemo Benefit, ICI Response	Independent prognostic factor; Improved ICI prediction over biomarkers; Identified non-responders in dMMR	Links imaging to biological TME state; International validation	(22)
Hao (2021)	Cox Model with ML Signatures (RF)	Clinical, CT Radiomics, CT Deep Learning Features	Intermediate/Late Fusion (Signatures + Cox)	Prognosis (OS, PFS) after Gastrectomy	C-index: 0.783 (OS), 0.770 (PFS); Imaging features added value to clinical/pathology	Compared pre- vs. post-operative models; Identified key features	(39)
Gao (2024)	iSCLM (Supervised Contrastive Learning)	CT Imaging, Biopsy WSI Pathology	Intermediate Fusion (Contrastive Learning)	Pathological Response to Neoadjuvant Chemo	AUCs: 0.846-0.876 (Test cohorts)	Highly interpretable via attention heatmaps, SHAP; Linked features to molecular pathology (CD11c)	(29)
Liu (2024)	Integrated DLR Model	CT, T2 MRI, DWI MRI (Traditional + DL Radiomics)	Feature Integration	Pathological Complete Response (pCR) to nCRT (ESCC, analogous)	AUC: 0.868 (Test)	Example from related GI cancer showing multi-modality imaging benefit	(41)
Tan (2023)	Radiopathomics Nomogram (SVM/MLP models)	CT Radiomics, WSI Pathomics	Late Fusion (Nomogram)	Staging (I-II vs III)	Radiomics AUC: ~0.77-0.80 (Test); Pathomics AUC: ~0.70-0.79 (Test); Integrated nomogram likely improves	Separate models for radiomics/pathomics; Integration via nomogram	(38)

See **Table 1** legend for abbreviations. DLR, Deep Learning Radiomics; DWI, Diffusion-Weighted Imaging; ESCC, Esophageal Squamous Cell Carcinoma; GNN, Graph Neural Network; IHC, Immunohistochemistry; iSCLM, Incremental Supervised Contrastive Learning Model; MuMo, Multi-Modal model; nCRT, Neoadjuvant Chemoradiotherapy; RF, Random Forest; SHAP, Shapley Additive Explanations; TME, Tumor Microenvironment.

Despite the promise, significant challenges remain in multi-modal AI. Acquiring complete datasets with all desired modalities for the same patient cohort can be difficult. Aligning data from different sources (e.g., mapping genomic alterations to specific regions on an image) is non-trivial. The computational cost and complexity of training these models are higher and ensuring the interpretability of how different data types contribute to the final prediction is crucial for clinical trust and adoption (36). Addressing the “black box” nature of complex models is vital. The development of interpretable multi-modal models, such as those using attention mechanisms (e.g., Grad-CAM for visualizing image focus) or Shapley values to quantify feature contributions, or those explicitly guided by biological principles (like BgDL or iSCLM linking predictions to TME or molecular features), represents a critical step towards clinical acceptance. By providing insights into the model’s reasoning process and linking predictions to recognizable biological or visual features, these approaches enhance transparency and facilitate validation.

Radiomics excels in non-invasive applications but struggles with generalizability, as seen in variable AUCs across regimens (28). Pathomics provides granular biological insights but is biopsy-dependent, limiting its use in serial monitoring. Multi-modal DL addresses these by synergizing modalities, consistently outperforming unimodal approaches (e.g., C-index gains in (39)), though it demands advanced XAI for clinical trust. Overall, multi-modal models represent the frontier but require prospective validation to mitigate data gaps (**Table 3**).

**Table 3:** Comparative analysis of key AI models in advanced gastric cancer.

Model Type	Strengths	Weaknesses	Best Applications in Advanced GC	Example Studies
Radiomics (e.g., feature extraction from CT/PET via ML like LASSO-LR, SVM)	Non-invasive; Uses existing imaging; Quantifies heterogeneity (e.g., texture features); High-throughput and cost-effective.	Dependent on image quality/standardization; Risk of overfitting due to high-dimensional features; Limited biological interpretability without XAI.	Metastasis detection (e.g., peritoneal); Response prediction (AUC 0.70-0.85); Longitudinal monitoring via delta-radiomics.	Li et al. (4); Jiang et al. (22)
Pathomics (e.g., DL on WSIs via ResNet, EfficientNet)	Captures microenvironment details (e.g., TILs, stroma); Objective biomarker quantification (e.g., PD-L1); Works on small biopsies common in advanced disease.	Requires digitized slides (not universal); Computationally intensive; Inter-scanner variability; Less effective for macroscopic features.	TIME analysis for ICI response (AUC >0.90); Subtype classification; Toxicity prediction potential.	Liu et al. (23); Gao et al. (29)
Multi-Modal DL (e.g., fusion of radiomics + pathomics + clinical via CNNs, GNNs, or contrastive learning)	Holistic integration reflects disease complexity; Superior performance (AUC improvements of 0.05-0.15 over unimodal); Models interactions (e.g., via attention mechanisms).	Data alignment challenges; Higher computational demands; “Black box” issues amplified; Scarce complete multi-modal datasets.	Personalized therapy selection (e.g., HER2+ICI); Prognosis in refractory settings; Adaptive models for resistance.	Zhou et al. (32); Jiang et al. (22); Gao et al. (29)

## 5 AI applications in refractory advanced gastric cancer

Patients with advanced GC whose disease progresses despite standard first- and second-line therapies face a particularly challenging situation with limited treatment options and a dismal prognosis (5). Identifying effective strategies for this refractory setting is a critical unmet need. AI offers potential avenues to address this challenge, although applications are currently more exploratory than established.

One potential role for AI is in understanding the mechanisms of treatment resistance. By analyzing longitudinal multi-modal data (e.g., serial imaging, sequential biopsies with genomic/transcriptomic profiling) from patients who develop resistance, AI algorithms might identify patterns or biomarkers predictive of failure. For example, identifying an epithelial-mesenchymal transition (EMT) phenotype, potentially detectable through imaging or molecular analysis, has been linked to resistance to both chemotherapy and immunotherapy in some cancers, and AI could help detect such signatures (42).

AI-driven drug repurposing represents another promising strategy (43). This involves using computational methods to identify existing drugs, approved for non-cancer indications, that may have anti-cancer activity in GC. AI algorithms can analyze vast databases encompassing drug structures, protein targets, gene expression profiles, pathway interactions, and scientific literature to predict potential drug-disease associations much faster than traditional screening methods. While numerous drugs have been investigated for repurposing in GC (e.g., metformin, statins, certain antibiotics, antidepressants), the use of AI specifically to identify these candidates for *refractory* GC is not yet widely documented in validated studies. However, the potential to rapidly screen existing compounds with known safety profiles makes this an attractive approach for finding new options quickly.^71^ This approach holds particular promise for refractory GC because it offers a potentially faster and less expensive route to finding effective therapies compared to the lengthy and costly process of *de novo* drug development.

Furthermore, AI could facilitate cross-cancer learning to generate treatment recommendations (44). Models trained on large datasets encompassing multiple cancer types might identify shared molecular vulnerabilities or predictive signatures for response to specific drugs, even if those drugs are not typically used in GC (45). This aligns with the concept behind basket trials like the NCI-MATCH study, which enrolls patients based on molecular alterations rather than tumor histology (46). However, the clinical utility of simply identifying an “actionable alteration” via NGS alone has been limited in advanced GC, with low rates of enrollment onto genotype-matched trials and uncertain clinical benefit. This highlights a potential key role for AI: moving beyond simple mutation matching to integrate genomic data with clinical context, imaging phenotypes, and pathway information, potentially learned from broader cross-cancer datasets, to more accurately predict the likelihood of benefit from a targeted therapy in a refractory patient (47, 48). AI might be a necessary component to effectively translate genomic findings into successful therapeutic strategies in the complex refractory setting.

Currently, these AI applications for refractory GC remain largely conceptual or in early research stages. Realizing their potential will require significant advancements in data infrastructure (integrated knowledge graphs, cross-institutional data sharing), robust predictive modeling capable of handling diverse data types, and validation in prospective clinical settings.

## 6 Bridging the gap: challenges, future directions, and clinical translation

Despite the significant progress and immense potential of AI in managing advanced GC, several substantial challenges must be addressed to bridge the gap between research findings and routine clinical practice.

### 6.1 Recap of key challenges

A recurring theme throughout the literature is the set of obstacles hindering the widespread adoption of AI tools. Data-related issues are paramount, including limitations in data quality, quantity, and heterogeneity across institutions. Lack of standardized data acquisition protocols (especially for imaging and digital pathology) and annotation methods makes it difficult to compare results and train generalizable models. Accessing large, well-curated, multi-modal datasets representing diverse patient populations remains a major hurdle, often leading to studies based on single-center, retrospective cohorts with limited external validity. Data privacy and security concerns also complicate data sharing efforts. Model-related challenges include the risk of overfitting to training data and poor generalizability to new, unseen data. The inherent “black box” nature of many complex DL models poses a significant barrier to clinical trust and adoption, as clinicians require transparency and understanding of how predictions are generated. Validation represents another critical bottleneck. The vast majority of studies rely on retrospective validation, with a striking lack of prospective trials evaluating AI tools in real-world clinical workflows. Standardized evaluation metrics and benchmarks are needed for objective comparison of different models. Finally, implementation challenges include integrating AI tools seamlessly into existing clinical decision support systems, navigating complex regulatory approval pathways (e.g., FDA clearance), and addressing ethical considerations surrounding algorithmic bias and accountability. The evidence suggests that the primary impediment to clinical translation may not be the sophistication of AI algorithms themselves, but rather these persistent issues surrounding data infrastructure and robust validation.

### 6.2 Future research directions

Addressing these challenges requires a multi-pronged approach. Data strategies should focus on fostering multi-center collaborations and establishing data sharing consortia to build larger, more diverse datasets. Implementing standardized protocols for data acquisition (e.g., imaging parameters, WSI scanning) and annotation is crucial. Techniques like federated learning, which allow models to be trained across multiple institutions without centralizing sensitive patient data, offer a promising solution to privacy concerns. Model development must prioritize robustness, generalizability, and interpretability. Continued research into Explainable AI (XAI) techniques is essential to build trust and facilitate clinical adoption. Advancing multi-modal fusion techniques to better capture synergistic information from different data sources remains a key area. Developing dynamic models that can incorporate longitudinal data (e.g., changes in imaging or biomarkers over time) could enable adaptive treatment strategies. Validation efforts must shift towards rigorous prospective clinical trials designed to assess the real-world impact of AI tools on clinical decision-making and patient outcomes. Head-to-head comparisons against current clinical standards and biomarkers are necessary. Specific needs for advanced GC include developing AI tools tailored for predicting response to novel combination therapies, identifying mechanisms of acquired resistance, guiding optimal therapy sequencing, improving toxicity prediction, and potentially incorporating patient-reported outcomes into predictive models.

### 6.3 Path to clinical translation

The journey from AI research to clinical practice requires careful navigation. Clear regulatory frameworks are needed to evaluate the safety and efficacy of AI medical devices. Successful implementation necessitates designing AI tools that are user-friendly and integrate smoothly into existing clinical workflows, providing actionable information to clinicians at the point of care. Education and training programs will be vital to equip clinicians with the knowledge and skills to effectively use and interpret AI-driven insights. Crucially, achieving meaningful clinical integration will demand a paradigm shift towards sustained interdisciplinary collaboration, bringing together oncologists, radiologists, pathologists, AI scientists, bioinformaticians, implementation scientists, ethicists, and regulatory bodies. This collaborative ecosystem is essential for ensuring that AI tools address genuine clinical needs, are rigorously validated, ethically deployed, and ultimately contribute to improved patient care.

## 7 Conclusion

Artificial intelligence is rapidly emerging as a transformative force in the management of advanced gastric cancer. Significant progress has been demonstrated in leveraging AI, particularly ML and DL techniques, to analyze complex data derived from radiomics, pathomics, genomics, and clinical records. These approaches show considerable promise in refining diagnostic accuracy relevant to treatment planning, predicting patient prognosis with greater precision, and, crucially, forecasting response to various systemic therapies including chemotherapy, immunotherapy, and targeted agents. The development of multi-modal AI models, capable of integrating information from diverse sources, represents a particularly important advancement, offering a more holistic understanding of tumor biology and patient characteristics, often leading to superior predictive performance compared to unimodal approaches. Furthermore, nascent applications of AI in identifying potential therapeutic avenues for refractory disease highlight its potential to address critical unmet needs.

The ultimate potential of AI lies in its ability to facilitate truly personalized medicine for patients with advanced gastric cancer. By uncovering subtle patterns hidden within complex data, AI can provide deeper insights into individual tumor biology, predict responses to specific treatments with greater accuracy, identify patients at high risk of toxicity, and optimize therapeutic strategies in ways previously unattainable.

However, the translation of this potential into routine clinical practice faces significant hurdles. Challenges related to data quality, accessibility, and standardization, coupled with the need for robust, prospective validation of AI models in diverse, real-world settings, remain major obstacles. Ensuring model interpretability and generalizability, navigating regulatory pathways, and achieving seamless integration into clinical workflows are also critical steps. Overcoming these challenges will require sustained, collaborative efforts involving researchers, clinicians, data scientists, industry partners, and regulatory agencies. Continued rigorous research focusing on high-quality data, robust validation, and the development of interpretable, clinically integrated AI tools is paramount. If these efforts are successful, AI holds the promise to fundamentally reshape the management of advanced gastric cancer, moving beyond empirical approaches towards a future of data-driven, personalized care that offers improved outcomes and hope for patients facing this challenging disease.
